# Minimally Invasive Necrosectomy Techniques in Severe Acute Pancreatitis: Role of Percutaneous Necrosectomy and Video-Assisted Retroperitoneal Debridement

**DOI:** 10.1155/2015/693040

**Published:** 2015-10-26

**Authors:** Jennifer A. Logue, C. Ross Carter

**Affiliations:** West of Scotland Pancreatico-Biliary Unit, Glasgow Royal Infirmary, 84 Castle Street, Glasgow G4 0SF, UK

## Abstract

Consensus advocating a principle of early organ support, nutritional optimisation, followed ideally by delayed minimally invasive intervention within a “step-up” framework where possible has radically changed the surgical approach to complications of acute pancreatitis in the last 20 years. The 2012 revision of the Atlanta Classification incorporates these changes, and provides a background which underpins the complexities of individual patient management decisions. This paper discusses the place for delayed minimally invasive surgical intervention (percutaneous necrosectomy, video-assisted retroperitoneal debridement (VARD)), and the rationale for opting to adopt a percutaneous approach over endoscopic or laparoscopic approaches in different clinical situations.

## 1. Introduction

The incidence of acute pancreatitis (AP) varies between populations ranging from 150 and 420 patients per million population in the UK to 330–430 patients per million in the USA [1, 2]. One in five patients, however, will develop organ failure with or without local complications–a setting that defines severe acute pancreatitis. Half of the deaths attributable to AP occur within the first 7 days of admission [3], with the majority in the first 3 days. Patients with severe AP who survive this first phase of illness, particularly those with persistent SIRS or organ failure [4], are at risk of developing secondary infection of pancreatic necrosis. Mortality in patients with infected necrosis and organ failure may reach 20–30% and an increased mortality is seen with increasing age [5]. The aim of this paper is to discuss the role of minimally invasive surgical intervention in severe acute pancreatitis, provide a rationale for adopting either a single or multimodality approach based on the often variable clinical scenarios, and highlight potential complications.

## 2. Revised Atlanta Classification 2012

The 2012 Revised Atlanta Classification [6] divides acute pancreatitis into three categories: mild, moderate, and severe disease. These categories are based on the absence or presence of local and/or systemic complications. In addition to disease severity, early mortality is strongly associated with age and comorbidity. Furthermore, the classification further categorizes local complications on the basis of time from presentation (< or > 4 weeks) and on the presence of necrosis (Table 1). The vast majority of acute fluid collections without necrosis will resolve within 4 weeks and a persistent fluid collection with minimal or no necrotic component (“pseudocyst”) is very rare. Collections may be sterile or infected. The majority of peripancreatic complications are therefore related to either acute necrotic collections (<4 weeks) or walled-off pancreatic necrosis (>4 weeks). This temporal separation is somewhat arbitrary, as the clinical management and surgical approach are determined by multifactorial individual patient factors. However, this does serve to provide a timeline beyond which, if appropriate, intervention should be delayed. A subsequent addendum added a category of “critical” recognizing those patients with sepsis and organ failure which was associated with the highest mortality [7].

## 3. “Step Up” Management of Postacute Peripancreatic Collections

Whilst the early management, rationale, timing, and technique of early percutaneous catheter drainage within a “step-up” framework have been discussed in previous sections, it is worth establishing the basis on which pancreatic necrosectomy may be considered within the minimally invasive era. Early debridement [8] has for many years been associated with and adverse outcome, in the absence of major (usually vascular) complications, being considered current standard practice. Freeny [9] and his colleagues in the 1990's showed that aggressive percutaneous sepsis control would promote recovery in the absence of formal necrosectomy, and this finding was confirmed within the PANTER trial [10] which demonstrated that 35% of patients with established necrotic collections did not require any further intervention over simple small diameter percutaneous catheter drainage.

Therefore, whilst a proportion of patients will recover without requirement for enhanced drainage, the majority will continue to exhibit signs of sepsis despite percutaneous catheter drainage alone. There is consensus that in those patients with persistent sepsis, a minimally invasive approach is preferred over open surgical necrosectomy, as described by Bradley, Warshaw, and Beger [11–13]. A number of “step-up” approaches have been described, including percutaneous necrosectomy (MIRP) [14], video-assisted retroperitoneal debridement (VARD) [15] and endoscopic [16] and laparoscopic [17] cystgastrostomy. Laparoscopic direct necrosectomy was described in the 1990's [18] but failed to gain popularity due to technical difficulty. There are 2 retrospective studies [19, 20] describing laparoscopic necrosectomy alone with a total of 29 patients. The patients were highly selected and no median follow-up was available for either study.

The choice of one approach over another is determined by the clinical condition of the patient, local experience and expertise, anatomical position/content of the collection, and the time from presentation/maturation of the wall of the collection. There is an acceptance that due to the complexity of presentation, no single technique is a panacea, and all options share a common concept of achieving minimally invasive sepsis control, whilst maintaining adequate nutritional competence. A detailed discussion surrounding nutritional support is beyond the scope of this paper but focuses on nasoenteric feeding [21] (NG or NJ), occasionally resorting to dual feeding (NJ/TPN) if nutritional targets are not being met enterically. Percutaneous gastrostomy or jejunostomy feeding tubes were commonplace in the open surgical era but are associated with procedure related complications which outweigh any advantage over the nasoenteric route and are not used in our unit.

The optimal approach is developing through evolution and the management concepts of the last decade, where solid predominant or infected necrotic collections were managed percutaneously by MIRP or VARD and well-organized predominantly fluid collections managed by endoscopic or laparoscopic transgastric drainage are now being challenged in randomized trials.

The choice of initial percutaneous or endoscopic drainage is now largely based on the position of the collection relative to the stomach, colon, liver, spleen, and kidney. Furthermore, the ability to perform EUS guided puncture within an ITU setting, without moving the patient to the radiology department for CT guided drainage, may be safer in a patient in extremis. In general, lateral collections and those extending behind the colon are usually better approached from the left or right flank whereas those medial collections where a percutaneous route is compromised by overlying bowel, spleen, or liver, may be better approached endoscopically. Initial percutaneous drainage is with an 8–12 FG single pigtail at the discretion of the radiologist, and catheter diameter or type does not seem to influence the requirement for secondary intervention. The route of percutaneous drainage should ideally take into account the probability of subsequent “step-up” escalation utilizing that drain tract, but the initial priority must be sepsis control, and if the initial drain placement is suboptimal, secondary alternative access can be obtained, sometimes involving a combination of percutaneous and endoscopic techniques.

**Figure 1:**
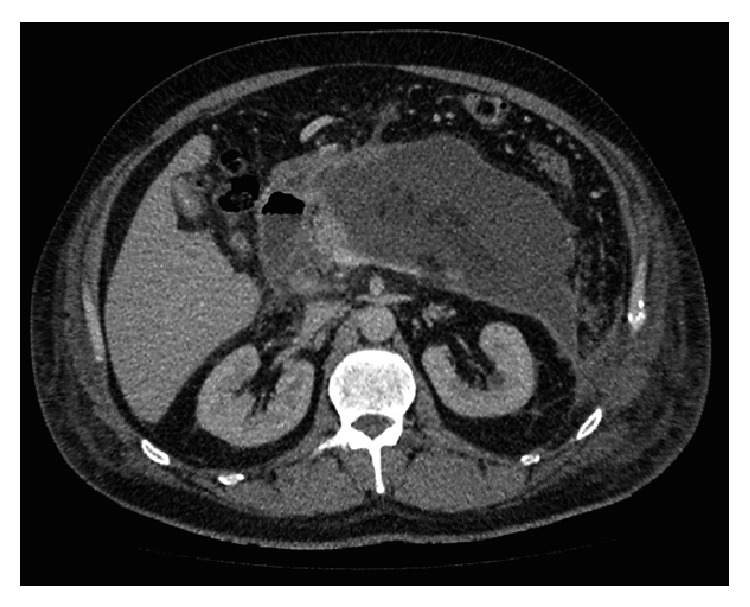
Acute walled-off pancreatic necrotic collection (W. O. P. N) at 6 weeks.

Both MIRP and VARD retroperitoneal techniques are modifications of the open lateral approach initially described in the 1980's by Fagniez et al. [22] which utilised a loin/subcostal and retrocolic approach to allow debridement of pancreatic and peripancreatic necrosis. This open approach was associated with major morbidity (enteric fistula 45%, haemorrhage 40%, and colonic necrosis 15%) and failed to gain popularity. For both minimally invasive techniques, a left-sided small diameter percutaneous drain is ideally placed into the acute necrotic collection between the spleen, kidney, and colon (Figure 1). Right-sided or transperitoneal drainage is also possible. In those who fail to respond adequately to simple drainage this access drain is then used as a guide to gain enhanced drainage of the collection.

**Figure 2:**
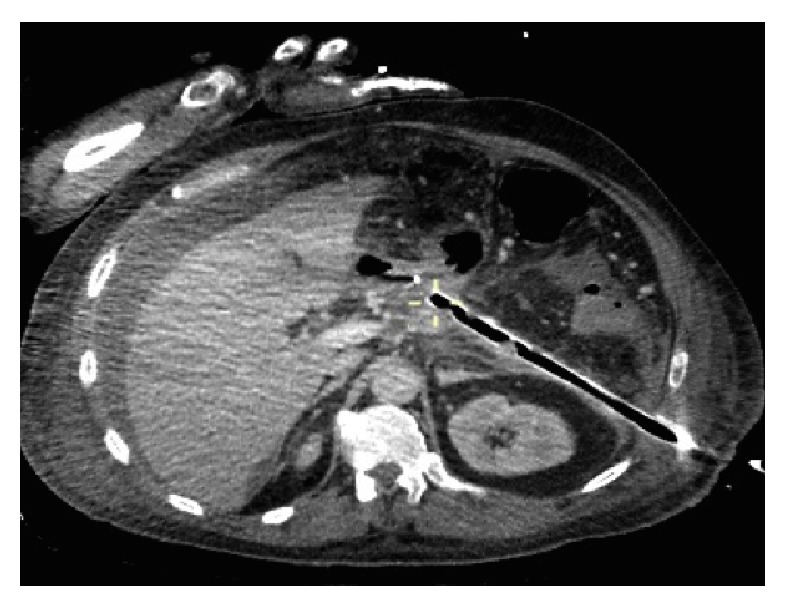
Retroperitoneal drain following enhanced “step-up” percutaneous necrosectomy in same patient as in Figure 1.

For percutaneous necrosectomy, the catheter is exchanged for a radiological guidewire and then a low compliance balloon dilator is inserted into the collection and dilated to 30 FG. Access to the cavity is achieved by passing the operating nephroscope through an Amplatz sheath, which allows debridement under direct vision. The nephroscope has an operating channel that permits standard (5 mm) laparoscopic graspers as well as an irrigation/suction channel. High flow lavage promotes initial evacuation of pus and liquefied necrotic material, exposing residual black or grey devascularised pancreatic necrosis and peripancreatic fat, which if loose is extracted in a piecemeal fashion until, after several procedures, a cavity lined by viable granulation tissue is created. At the end of the procedure an 8 FG catheter sutured to a 24 FG drain is passed into the cavity to allow continuous postoperative lavage of warm 0.9% normal saline initially at 250 mls an hour (Figure 2). The subsequent rate of lavage is determined by the return and can be reduced as the effluent clears and the clinical control of sepsis is achieved. Chemically assisted debridement with hydrogen peroxide has been reported during endoscopic drainage [23], but concerns regarding the risk of air embolism have been highlighted in previous studies [24] and should only be considered within a study format. Subsequent conversion of the lavage system to simple drainage may be all that is required prior to recovery or the procedure may be repeated until sepsis control is achieved and interval CT confirms resolution.

A video-assisted retroperitoneal debridement (VARD) procedure is performed with the patient placed in a supine position with the left side 30–40° elevated. A subcostal incision of 5 cm is placed in the left flank at the midaxillary line, close to the exit point of the percutaneous drain. Using the* in situ* percutaneous drain as a guide the retroperitoneal collection is entered. The cavity is cleared of purulent material using a standard suction device. Visible necrosis is carefully removed with the use of long grasping forceps, and deeper access is facilitated using a 0° laparoscope, and further debridement performed with laparoscopic forceps under videoscopic assistance. As with a percutaneous necrosectomy, complete necrosectomy is not the aim of this procedure and only loosely adherent pieces of necrosis are removed, minimizing the risk of haemorrhage. Two large bore single lumen drains are positioned in the cavity and the fascia closed to facilitate a closed continuous postoperative lavage system.

## 4. Early Procedure Related Complications

### 4.1. SIRS/Bacteraemia Requiring Critical Care Support

Occasionally intervention is associated with a significant SIRS or postprocedure bacteraemia, requiring critical care admission for organ support and often vasopressor therapy. This often resolves within 24–48 hours with appropriate supportive management. Therefore if possible, it is often beneficial for these patients to be observed in a critical care environment following intervention, particularly if they were not receiving level two care before procedure.

### 4.2. Acute or Delayed Haemorrhage

Probably the most frequent scenario is brisk haemorrhage complicating early or overenthusiastic necrosectomy. Attempts at open surgical haemostasis are associated with significant mortality, and in this setting control is usually achieved by packing, or balloon tamponade, but emergency angiography may occasionally have to be considered for arterial bleeding. Venous bleeding is common and should be suspected in patients with a nondiagnostic angiogram but usually settles with correction of any coagulopathy and with local pressure, by simple drain occlusion, a modified Sengstaken-Blakemore tube having amputated the gastric balloon (MIRP), or gauze packing if there is sufficient cutaneous access (VARD).

Secondary haemorrhage is occasionally sudden and massive, but there is usually a prelude with a “herald bleed,” a self-terminating bleed presenting clinically with haemorrhage into a retroperitoneal drain or occasionally a gastrointestinal bleed. An arterial origin of haemorrhage is more common than venous when this occurs as a spontaneous secondary bleed. Overall, the mortality exceeds 30–40% and a high index of suspicion is essential in order to optimise proactive treatment. In our unit the patient is rapidly stabilized with controlled volume support of the circulation and a simultaneous emergency CT angiogram. Upper gastrointestinal endoscopy in this setting is usually nondiagnostic so should not delay radiological assessment which allows definitive management. Where arterial bleeding is identified on formal angiography embolisation offers the best chance of survival. The increased intracavity pressure, associated with haemorrhage into an infected cavity, may also often be followed by escalating organ dysfunction through bacteraemia and sepsis; therefore early introduction of targeted antimicrobials is essential.

### 4.3. Enteric Fistulation

Spontaneous discharge of a postacute collection into the gastrointestinal tract is also recognised which can decompress the collection and result in a clinical improvement without intervention, usually where the fistulous communication involved is the stomach or duodenum, mimicking an endoscopic drainage. It can also present with haematemesis or melaena and should be managed as described above. Whilst spontaneous resolution is possible, fistulation into the colon will often result in persistent sepsis and poorly controlled collections, and therefore, in this situation a defunctioning colostomy/ileostomy or resection may be necessary.

### 4.4. Late Complications

#### 4.4.1. Pancreatic Fistulation

Parenchymal necrosis is commonly associated with disruption of the main pancreatic duct, and following resolution of associated sepsis residual duct leakage of amylase rich fluid is common, leading to a pancreatic fistula. Early endoscopic intervention should be discouraged whilst collections remain as this may introduce infection and usually prove detrimental. Following resolution of sepsis and any significant collection, transpapillary pancreatic duct stent insertion at ERCP may result in resolution of a persistent fistula, but persistent drainage is often associated with more extensive parenchymal loss or a disconnected tail (see below). Prolonged catheter drainage will lead to maturation of the fistula tract and interval drain removal may result in spontaneous resolution or development of a postacute pseudocyst, which can often be resolved by transmural endoscopic cystgastrostomy achieving enteric diversion.

#### 4.4.2. Disconnected Tail

Where the necrosis extends across most of the transverse diameter of the body or tail, complete separation of the main pancreatic duct in the head of the pancreas and tail may occur leading to a persistent fistula and “disconnected duct syndrome.” Ductal occlusion at the level of parenchymal loss often precludes transpapillary access but if this has not occurred, intracystic transpapillary stenting may result in resolution. If transpapillary access is not possible, options may be transmural EUS guided drainage. Surgical options are dependent on the anatomy and degree of anatomic distortion following resolution of the early phase of disease [25] and include a formal pancreaticojejunostomy, fistula-jejunostomy, or a “salvage” distal pancreatectomy to excise the residual disconnected functional pancreatic parenchyma, often in combination with a splenectomy, performing a limited pancreatico- (fistula-) jejunostomy.

## 5. Discussion

There is general agreement that intervention in the first two weeks of severe AP should be avoided if at all possible. During this period, many patients may require intensive care management, with escalating organ failure associated with a significant mortality [4], but intervention for the pancreatic or peripancreatic inflammatory mass has not been shown to enhance recovery and may be detrimental. Rare exceptions to the noninterventional approach include the presence of intra-abdominal haemorrhage or necrosis of bowel. In either case, it is better, if possible not to disturb the pancreatic inflammatory mass at this time. Thus, pancreatic intervention should be delayed until walled-off necrosis has developed, typically 3–5 weeks after onset of symptoms. Several observational studies have shown improved outcome for operation beyond 28 days from onset [26]. Some authors have expressed concern that delay beyond this risks the patient's general condition which will deteriorate, with resultant impaired nutritional and immune status, but where slow improvement continues, delay until established WOPN simplifies intervention.

The role of antibiotics has evolved in the last 20 years from a position supporting prophylaxis to one of selected targeted administrations to manage proven episodes of infection, with positive blood cultures or radiological evidence of infection [27]. Furthermore, attempts to confirm infection of necrosis by fine needle aspiration [28] of collections are no longer favored and treating positive drain culture in the absence of clinical sepsis results in emergence of fungal overgrowth or antibiotic resistance. Antibiotics may facilitate a delay in definitive intervention for infected necrosis presenting within the first 4 weeks, at which stage intervention is aimed at sepsis control.

Indications for intervention include strong suspicion or documented infection of necrosis or in the absence of infection, persistent organ failure for several weeks, with a walled-off collection and persistence of symptoms such as pain and ileus. Timing of intervention requires a judgment call by an experienced and specialist pancreatic team, involving a multifactorial decision algorithm based on radiological, clinical, and nutritional progress. Once the decision for intervention has been made, the options include open surgical necrosectomy, percutaneous or other forms of minimally invasive surgical necrosectomy, percutaneous catheter drainage, and endoscopic necrosectomy through the stomach as described above. No single treatment approach is ideal for use in all patients, and in practice a range of options may be required, often in combination, based on the position of the acute necrotic or walled-off collection taken in context with the patient's overall clinical condition.

There have been a number of studies attempting to compare types of intervention in acute pancreatitis. The complexity of presentation and evolution of the disease process, the relative position and content of collections, and relative rarity of these patients have made large scale trials with homogeneous characteristics impossible. Despite the challenges some small-scale studies have been completed which have informed the debate. The PANTER study [10], by the Dutch Pancreatitis study group, demonstrated an advantage to a minimally invasive approach (VARD) over open necrosectomy in early postprocedural organ dysfunction within a “step-up” management algorithm. The study was not powered to consider mortality, and perhaps the most significant finding was that 35% of patients resolved completely with small diameter catheter drainage alone. Other pilot studies by this group have explored the potential of endoscopic transmural drainage versus minimally invasive intervention (VARD), the PENGUIN trial [29] suggesting at least equivalence, if not benefit, from endoscopic drainage, but this has been criticized due to very small numbers and an excessive mortality, compared to the groups historical results, within the VARD arm. The results of the resultant TENSION trial [30] are awaited with interest.

Minimally invasive approaches have been criticized as they often require repeated intervention prior to resolution, with increased inpatient stay. In a clinically well patient with established walled-off necrosis, whose principal symptom is failure to thrive, a laparoscopic transgastric cystgastrostomy offers the potential of a single intervention with the possibility of simultaneous definitive management of cholelithiasis [17]. Worhunsky et al. recently reported a series of 21 patients [31] with retrogastric pancreatic necrosis who underwent debridement with a single intervention using laparoscopic transgastric necrosectomy suggesting that where feasible this allows primary definitive management. Cyst content (whether the acute necrotic collection/walled-off necrosis was predominantly fluid or solid) traditionally influenced decision-making; however limiting endoscopic transmural drainage to only fluid predominant collections has been challenged with increasing experience of endoscopic necrosectomy. We are currently engaged in a randomised trial of laparoscopic versus endoscopic cystgastrostomy in patients with walled-off necrosis and the results are awaited.

Complications following enhanced drainage are common and may be either disease or procedure related. Enteric fistulation is relatively common, and the requirement for secondary control is dependent on whether the fistula arises from the proximal or distal gut, colonic fistulae often requiring surgical enteric diversion to control persistent sepsis. Bleeding may occur intraoperatively and may be controlled by balloon tamponade, conversion to a VARD procedure with gauze packing, or occasionally angiography. Venous bleeding is more common intraoperatively. Secondary haemorrhage may arise on the background of poorly controlled sepsis and in the presence of an enteric fistula may result in GI bleeding or direct bleeding within a surgical drain. Angiographic control or again local pressure via the drain tract or VARD wound is preferred to open surgery, which historically was often an agonal intervention.

There is consensus that, within a “step-up” environment, some form of minimally invasive approach is superior to open intervention particularly in the critically ill patient. Operator experience is a key determinant of which minimally invasive approach to adopt. There is no evidence supporting the use of one approach over another. The VARD approach utilizes standard laparoscopic and surgical instruments whilst a minimally invasive necrosectomy utilizes standard urological equipment both of which are universally available. Many units may have experience in only one method, and this will influence the decision process. The differences between a VARD and MIRP are small, and in practice these procedures are interchangeable, whereas the addition of either an endoscopic or laparoscopic cystgastrostomy can increase management options particularly where collections are centrally placed and percutaneous access is difficult. A “gold standard” minimally invasive management algorithm would take into account the clinical condition of the patient, anatomical location of the collection and in an ideal world expertise in all 4 techniques which allows for adaptability and flexibility in the interventional approaches to an often extremely challenging clinical problem. An important point to note is that many patients may benefit from the use of a multimodal approach with the use of more than one technique during the course of their illness. For example a patient with escalating multiorgan failure can be stabilized within the ICU setting with EUS guided transgastric drainage and following a period of stabilisation more definitive intervention employed by either MIRP, VARD, or even laparoscopic cystgastrostomy. In reality, however, most units will not necessarily have access to all techniques which will obviously impact on management decisions.

In conclusion, in a patient with established criteria for intervention, simple percutaneous (or endoscopic) drainage of the dominant collection is indicated. Careful subsequent clinical observation with monitoring of biochemical and haematological indices will determine whether enhanced drainage is required, in which case where initial percutaneous catheter drainage was the initial procedure, a minimally invasive necrosectomy or VARD, establishing a postoperative continuous closed lavage system, will improve sepsis control and optimise outcome and the procedure may be repeated as required. The results of a number of randomised studies are awaited to inform the debate as to the optimal choice of enhanced surgical or endoscopic intervention within a step-up environment.

## Figures and Tables

**Table 1:** Local complications in acute pancreatitis (2012 Revised Atlanta Classification).

Time scale	Necrosis absent	Necrosis present
<4 weeks	Acute peripancreatic fluid collection (peripancreatic fluid associated with interstitial oedematous pancreatitis with no associated peripancreatic necrosis)	Acute necrotic collection (a collection containing variable amounts of both fluid and necrosis; the necrosis can involve the pancreatic parenchyma or the extrapancreatic tissues)

>4 weeks	Pancreatic pseudocyst (an encapsulated collection of fluid with a well-defined inflammatory wall usually outside the pancreas with minimal or no necrosis)	Walled-off necrosis (a mature, encapsulated collection of pancreatic or extrapancreatic necrosis that has developed a well-defined inflammatory wall)

Infection	Each collection type may be sterile or infected	
